# Comparison of incidence and outcome between occupational and non-occupational motorcycle injuries in Korea: A 7-years observational study

**DOI:** 10.1371/journal.pone.0283512

**Published:** 2023-03-29

**Authors:** Sungbae Moon, Hyun Wook Ryoo, Jae Wan Cho, Haewon Jung, Kang Suk Seo, Kyoung Hoon Lim

**Affiliations:** 1 Department of Emergency Medicine, School of Medicine, Kyungpook National University, Daegu, Republic of Korea; 2 Department of Surgery, School of Medicine, Kyungpook National University, Daegu, Republic of Korea; Zhejiang University, CHINA

## Abstract

Motorcycles are widely used in various workplaces. Motorcycle use for occupational purposes continues to increase owing to growing e-commerce. Here, we aimed to highlight the importance of occupational motorcycle injuries by analyzing their epidemiologic characteristics and outcomes. We analyzed retrospective data from the Emergency Department-based Injury In-depth Surveillance program from 2012 to 2018. Motor vehicle injuries involving riders aged ≥16 years were included. Patients were divided into occupational motorcycle and non-occupational motorcycle injury groups based on whether or not the injury occurred during work time. General characteristics, injury details, and clinical outcomes such as injury severity and in-hospital mortality were analyzed. Of the 37,194 study patients, 24.2% (8,991) experienced occupational motorcycle injuries. The number of injuries in both groups increased yearly, as did the proportion of occupational injuries among total injuries. In both the groups, patients aged 20–29 years had the highest proportion of injuries. Regarding collision pattern and injury counterpart, side-to-side collisions and injuries involving small four-wheel vehicles were the most frequent. Alcohol intake was significantly lower, while helmet usage was higher in the occupational motorcycle injury group. Moreover, patients with occupational motorcycle injuries had lower injury severity, admission rate, and in-hospital mortality. On multivariable logistic regression analysis, increasing age, time of the injury, alcohol intake, not using a helmet, and collision with a human or animal were associated with higher odds of severe injury. Patients with occupational injuries had higher helmet usage, lower injury severity, lower mortality, and lower admission rate than did patients with non-occupational injuries. Injury severity was associated with the time of injury, collision with other living objects, alcohol consumption, and helmet usage.

## Introduction

Nowadays, motorcycles are widely used in various workplaces. With the continuous rise of online commerce, the delivery industry and courier services continue to grow [1, 2]. Moreover, changes in lifestyle, such as increased solitary living, particularly in developed countries with densely populated metropolitan areas, have contributed to increased reliance on deliveries and courier services. Furthermore, the coronavirus disease pandemic triggered an increase in the demand for deliveries worldwide [2–4]. In turn, this has led to the widespread use of motorcycles for occupational purposes. Therefore, occupational motorcycle injuries (OMIs) are gaining attention, as injuries to motorcycle riders affect their daily life. Additionally, an increase in OMIs poses a burden to both service providers and patients [5]. From a medical perspective, motorcycles are usually more mobile and compact than cars; hence, they can lead to more severe injuries and increased rider mortality than do typical vehicle accidents [6]. Therefore, it is imperative to analyze the patterns of OMIs, an important social and medical phenomenon that needs to be evaluated and addressed.

Although OMIs are gaining public and academic interest, only a few studies have been conducted on this topic. Studies evaluating motorcycle injuries tend to focus on certain age groups [7], a specific geographic location [8], passengers as a passenger service [9], or recreational motorcycle use [10]. The few studies that have evaluated OMIs are either surveys or interviews [11–14]; alternatively, these studies have focused on non-medical factors [14–18] or certain disease entities [19, 20].

Here, we aimed to highlight the impact of OMIs by analyzing their epidemiologic characteristics and determining factors associated with severe injury using a multicenter nationwide database.

## Materials and methods

### Study design and subjects

This was a retrospective cross-sectional study. Data were collected through the Emergency Department-based Injury In-depth Surveillance (EDISS) program. EDISS data from 2012 to 2018 were used in this study [21]. The surveillance program is managed by the Korean Disease Control and Prevention Agency (KDCA) and was started in 2005. This program aims to collect clinical and statistical data on injured patients visiting emergency departments (EDs). Such data is used to promote injury prevention. Presently, 23 hospitals in Korea take part in the surveillance and data collection network. Designated coordinators in each participating hospital collect pre-hospital data from emergency medical service (EMS) records and clinical data from hospital medical records. If needed, coordinators conduct face-to-face or telephone interviews to supplement missing variables. The collected data are periodically uploaded to the EDISS data registry system operated under the Integrated Disease & Health Management System and managed by the KDCA. Access to EDISS data may be granted with permission of KDCA.

Of the EDISS data collected during the study period, only motor vehicle injuries involving motorcycle drivers were selected and included in this study. This was done by selecting ‘Motorcycle’ under ‘Motor vehicle accident’ in an EDISS variable named ‘Injury mechanism,’ which includes all collisions and injuries involved with motor vehicles. In addition, another EDISS variable named ’Patient role at the vehicle accident’ specifying the type of passenger status was used to select motorcycle drivers. Furthermore, only patients aged ≥16 years were included. Patients with an unclear age, vehicle type, driver-rider status, pre-hospital data, or incomplete in-hospital clinical data were excluded. Patients were divided into OMI and non-OMI groups. The definition of “occupational” injury was determined by ’Activity during injury’ in the EDISS variable which also collects information regarding occupational relevance. On data collection and recording, cases were defined to be occupational if the injury occurred ’During work’ or ‘During economic and occupational activity’.

**Fig 1:**
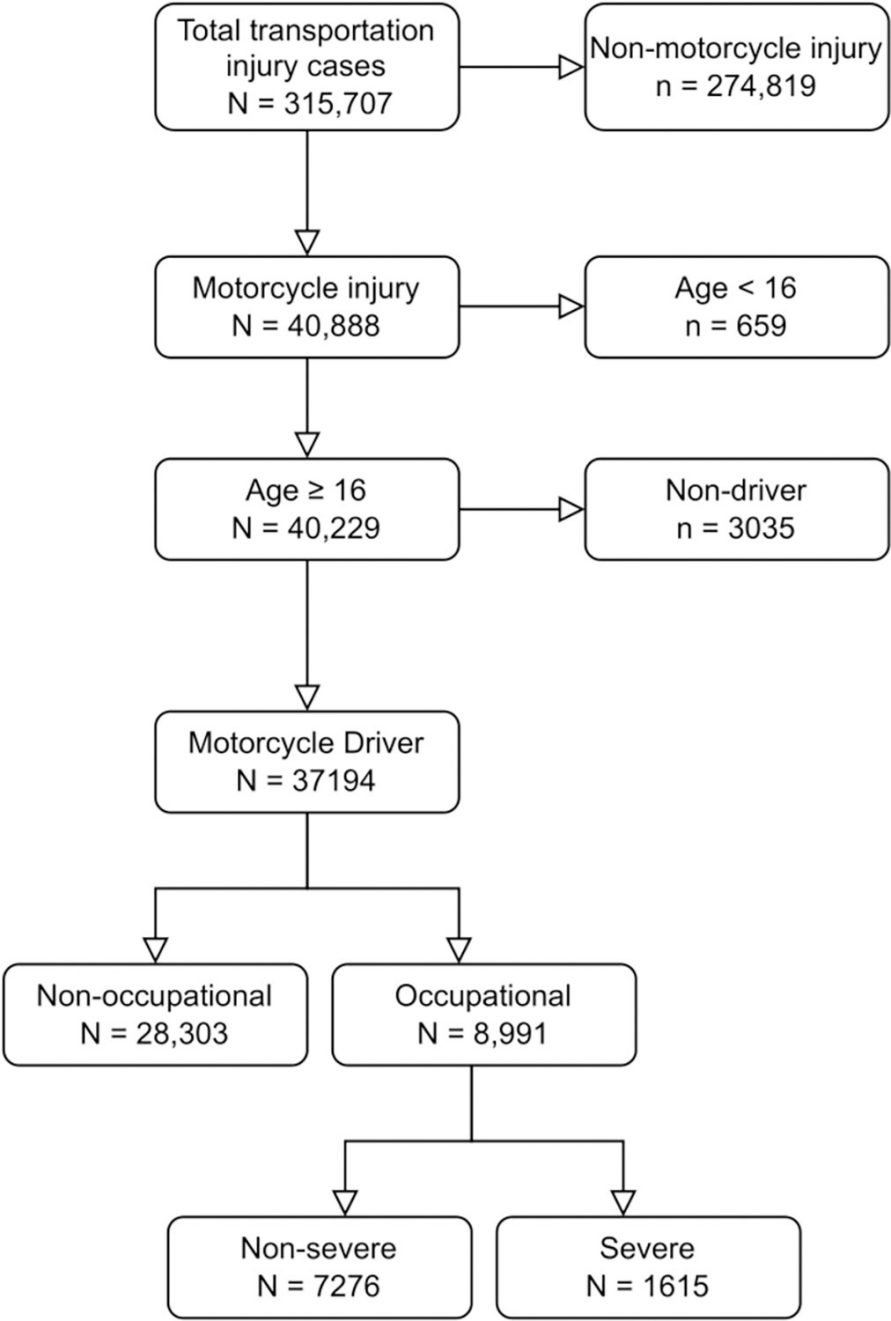
Flow of motorcycle injury cases included in this study.

During the study period, a total of 315,707 patients visited the EDs for transportation injuries. After applying the study inclusion and exclusion criteria, 37,194 patients were included in this study. The flow of patients, including reasons for exclusion at various steps, is shown in Fig 1.

### Study variables

From the EDISS database, to investigate and compare the injury characteristics and clinical outcome of OMIs from non-OMIs, general characteristics (such as patient age, sex, car insurance status, ED arrival route), injury details (including injury year, time, place, collision pattern, injury counterpart, alcohol consumption, and helmet use), and clinical outcomes such as injury severity, traumatic central nervous system injury, facial injury, and length of stay, and overall hospital mortality were selected and analyzed. Age groups were divided by 10 years, except for the elderly, defined as those aged ≥65 years by Road Traffic Act in Korean legislation. Injury time was divided into three categories; day (7:01 AM to 6:00 PM), evening (6:01 PM to 12:00 PM), and night (12:00 AM to 07:00 AM). Insurance was based on what kind of reimbursement method the patients used, such as national health insurance, car insurance, industrial injury compensation as work insurance, or no insurance at all. In EDISS, acquiring injury details mostly depended on a statement made by patients, companions, or paramedics involved in patient transport. This policy was also applied to the alcohol consumption of the drivers since Korean emergency medical practice and current EDISS data collection guidelines do not mandate ordering blood alcohol tests as a routine. Collision pattern was defined as head-on, side-to-side collisions, rear-end, capsized, or a combination of such collision patterns. Injury counterpart vehicles were classified as bicycles or motorcycles with less than four wheels, small-sized passenger vehicles with four more wheels and capable of transporting lesser then 20 passengers, including minibus or pick-up trucks, large/heavy commercial vehicles, other transportation methods such as train or monorails, fixed stationary objects, a living organism such as human or animals, or no counterpart if there were no collisions occurred such as driver self-collapsing or ejecting from the motorcycle. Injury severity was measured using the Excess Mortality Ratio-adjusted Injury Severity Score (EMR-ISS). The EMR-ISS system is a measuring tool used to determine the severity of injury in patients and has some advantages over the conventional Injury Severity Scoring system [22]. Patients with an EMR-ISS higher than 24 points were classified as having severe injuries. Traumatic head injury included an open wound or fracture of the head, any form of intracranial hemorrhage, edema, diffuse axonal injury, or crushing injury to the head. Traumatic neck injury included injury of vertebrae, vertebral joints and ligaments, intervertebral discs, spinal cord, or spinal nerve roots. Overall mortality was defined as patient’s death within 30 days after visiting ED. The primary outcome of this study was injury severity.

### Statistical analysis

In comparing OMI and non-OMI cases, continuous variables were summarized using the median and interquartile range and were compared using the t-test, while categorical variables were presented with frequency and percentage and were compared using the chi-square test. *P*-values < 0.05 were considered statistically significant. The same analysis was made to compare the characteristics between severe OMI and non-severe OMI cases.

Logistic regression analyses were used to determine the factors associated with severe injury among patients with OMIs. Adjusted odds ratios (AORs) and 95% confidence intervals (CIs) were computed after adjusting for age, sex, injury time of the day, ED arrival method, the place of the injury, collision pattern, and the injury counterpart.

Statistical analyses were performed using SAS software, Version 9.4 (SAS Institute, Cary, NC, USA).

### Ethics statement

This study was approved by the Kyungpook National University Hospital Institutional Review Board. The requirement for informed consent from the participants was waived owing to the retrospective study design.

## Results

**Fig 2:**
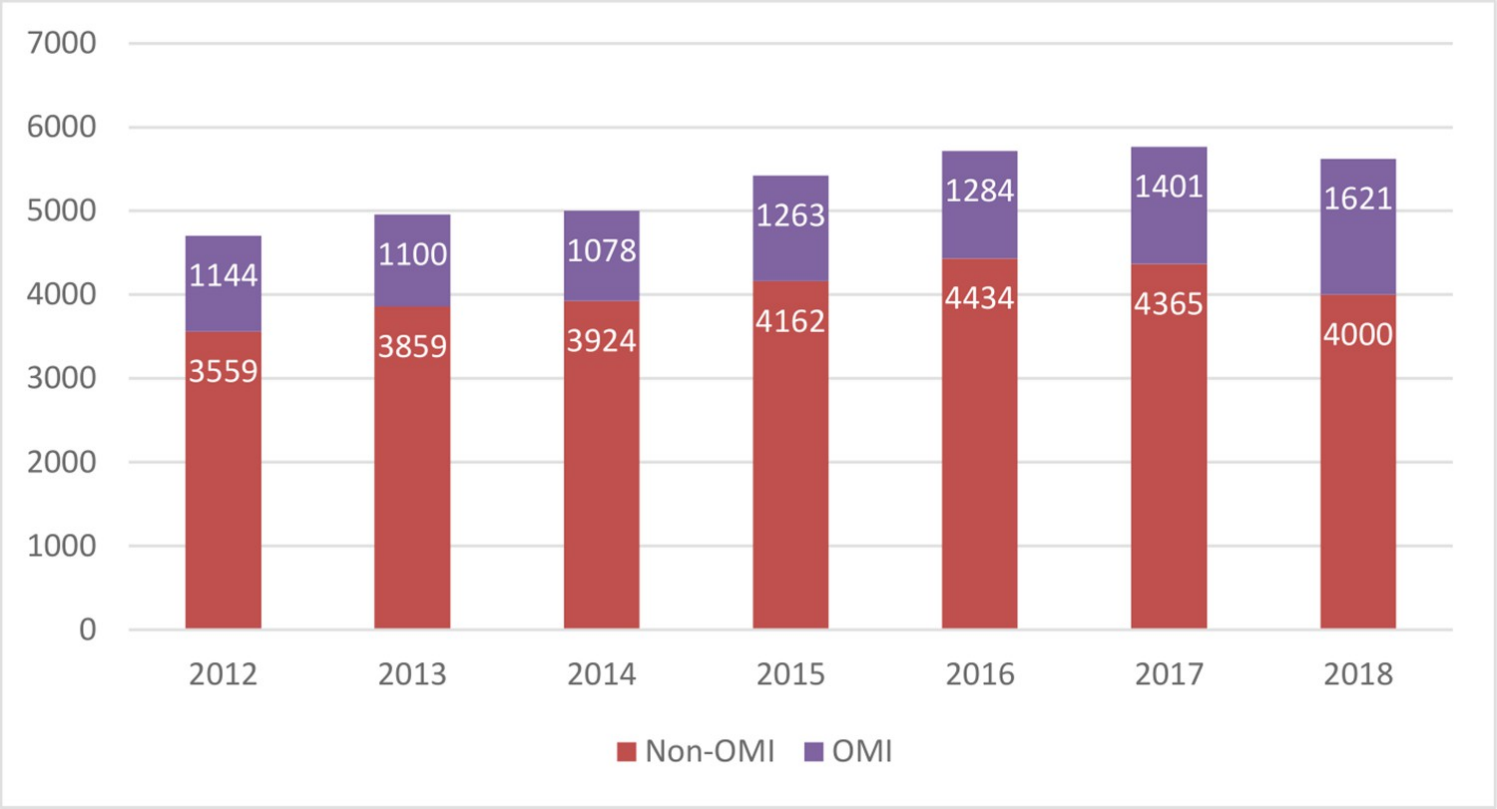
Analysis of motorcycle injury cases by year. OMI; occupational motorcycle injury.

**Fig 3:**
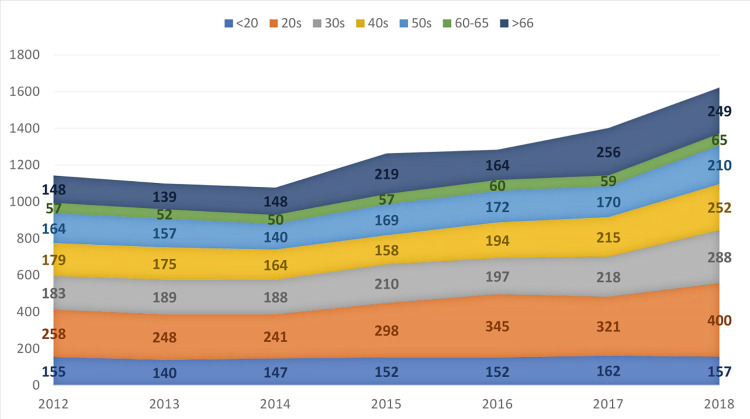
Analysis of occupational motorcycle injury according to year and victims’ age group.

**Fig 4:**
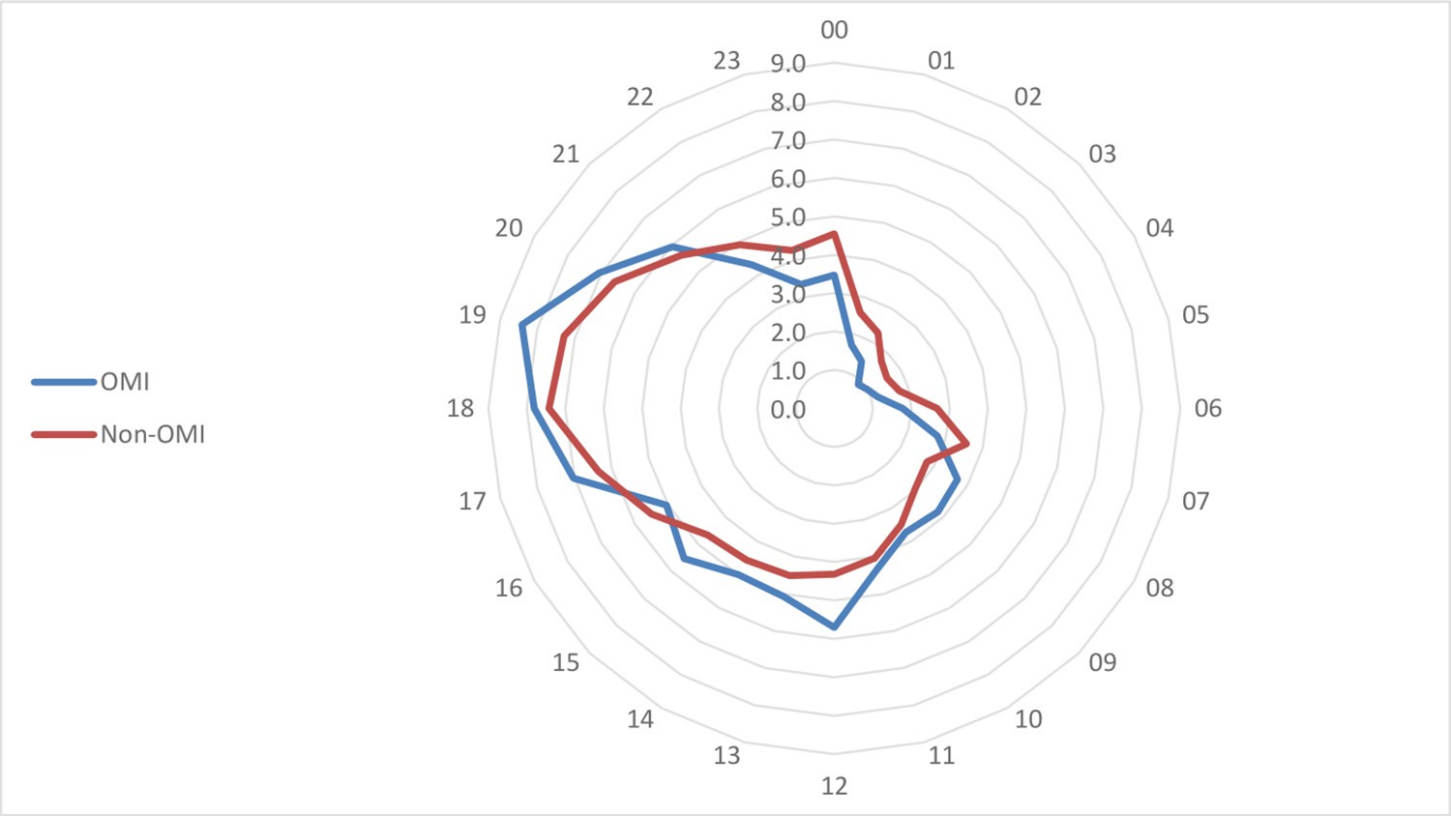
Comparison of OMI and non-OMI according to injury time. OMI; occupational motorcycle injury.

The general and demographic characteristics of study participants are shown in Table 1. As shown in Fig 2, there was an increase in both OMIs and non-OMIs annually apart from 2014. Detailed age groups and hourly analyses are shown in Figs 3 and 4. After dividing the time of day into dawn, day, and night, OMIs were relatively less frequent during the night hours. Regarding the type of medical insurance, the percentage of patients whose medical expenses were covered using car insurance was significantly higher in the OMI than in the non-OMI group.

**Table 1:** General characteristics, demographic characteristics, injury details, and clinical results of motorcycle injury cases in the study period, according to the occupational or non-occupational environment.

		Non-OMI (N = 28303)		OMI (N = 8891)		Total (N = 37194)		
		N	%	N	%	N	%	p-value
Age	Median (Q1-Q3)	34 (22–56)		38 (24–55)		35 (23–56)		<0.001
Age group	<20	5206	18.4	1065	12.0	6271	16.9	
	20s	6607	23.3	2111	23.7	8718	23.4	
	30s	4228	14.9	1473	16.6	5701	15.3	
	40s	3051	10.8	1337	15.0	4388	11.8	
	50s	3152	11.1	1182	13.3	4334	11.7	
	60–65	1259	4.4	400	4.5	1659	4.5	
	>65	4800	17.0	1323	14.9	6123	16.5	
Sex	Male	26435	93.4	8194	92.2	34629	93.1	<0.001
	Female	1868	6.6	697	7.8	2565	6.9	
Injury time of the day	Day (07–18)	13228	46.7	4596	51.7	17824	47.9	<0.001
	Evening (18–24)	10221	36.1	3280	36.9	13501	36.3	
	Night (00–07)	4854	17.2	1015	11.4	5869	15.8	
ED arrival route	119 ambulance	15783	55.8	5204	58.5	20987	56.4	<0.001
	Other ambulance	4734	16.7	1085	12.2	5819	15.6	
	Foot or own car	7594	26.8	2552	28.7	10146	27.3	
	Other transport	192	0.7	50	0.6	242	0.7	
Insurance	National Health	8694	30.7	2536	28.5	11230	30.2	<0.001
	Car	15897	56.2	5723	64.4	21620	58.1	
	Industrial injury Compensation	73	0.3	36	0.4	109	0.3	
	Noninsured	3564	12.6	586	6.6	4150	11.2	
	Other	75	0.3	10	0.1	85	0.2	
Place	Highway/motorway	499	1.8	442	5.0	941	2.5	<0.001
	Common road	25345	89.5	7370	82.9	32715	88.0	
	Alley/farm road	1764	6.2	892	10.0	2656	7.1	
	Other	695	2.5	187	2.1	882	2.4	
Collision pattern	Head on	2797	9.9	730	8.2	3527	9.5	<0.001
	Side-to-side	2263	8.0	749	8.4	3012	8.1	
	Rear-end	902	3.2	164	1.8	1066	2.9	
	Capsized	1813	6.4	566	6.4	2379	6.4	
	Combination	391	1.4	351	3.9	742	2.0	
	Other	1552	5.5	247	2.8	1799	4.8	
Injury counterpart	Bicycle/motorcycle	851	3.0	315	3.5	1166	3.1	<0.001
	Small vehicles, ≥ four wheels	11469	40.5	4412	49.6	15881	42.7	
	Large vehicles	975	3.4	382	4.3	1357	3.6	
	Fixed object	1091	3.9	237	2.7	1328	3.6	
	No counterpart	5942	21.0	1723	19.4	7665	20.6	
	Human/animal	184	0.7	68	0.8	252	0.7	
	Other	621	2.2	132	1.5	753	2.0	
Alcohol	Yes	2068	7.3	263	3.0	2331	6.3	<0.001
Helmet	Yes	13976	49.4	5655	63.6	19631	52.8	<0.001
Severity		6438	22.7	1615	18.2	8053	21.7	<0.001
EMR-ISS	Median (Q1-Q3)	10.0 (8.0–25.0)		9.0 (4.0–22.0)		10.0 (6.0–25.0)		<0.001
TBI or TSI or facial injury		1498	5.3	1370	15.4	2868	7.7	0.044
TBI		1055	3.7	697	7.8	1752	4.7	<0.001
TSI		291	1.0	375	4.2	666	1.8	<0.001
ED LOS (hour)	Median (Q1-Q3)	3.1 (1.6–6.1)		3.0 (1.6–5.7)		3.1 (1.6–6.0)		<0.001
Admission		9622	34.0	2628	29.6	12250	32.9	<0.001
Hospital LOS (day)	Median (Q1-Q3)	14.2 (7.5–25.1)		13.4 (7.0–23.7)		14.0 (7.4–24.8)		0.014
Overall mortality + hopeless discharge		877	3.1	156	1.8	1033	2.8	<0.001

OMI; occupational motorcycle injury, ED; emergency department, EMR-ISS; excess mortality ratio-adjusted injury severity score, TBI; traumatic brain injury, TSI; traumatic spine injury, LOS; length of stay. ‘119 ambulance’ is the national EMS service in the Republic of Korea. Note that severity is determined by EMR-ISS over 24 points.

The details of motorcycle injuries are also shown in Table 1. The rate of alcohol consumption in the OMI group was 3.0%, compared to 7.3% in the non-OMI group. Regarding protective equipment, a significantly higher proportion of occupational drivers wore helmets (63.6% vs. 49.4%) than did non-occupational drivers. Regarding colliding patterns with other vehicles, side-to-side collision (8.4%) was the most common colliding pattern among patients with OMIs. Small vehicles with four or more wheels were the most common injury counterpart (49.6%, 40.5%), regardless of occupational injury status.

In variables associated with severity, the median EMR-ISS score significantly differed (9.0 vs. 10.0) between the OMI and non-OMI groups, and the rate of severe trauma injuries was lower (18.2% vs 22.7%) in patients with OMIs than in those with non-OMIs. The traumatic brain, spine, or facial injury rate was higher among patients with OMIs than among patients with non-OMIs (15.4% vs. 5.3%). There were fewer admissions in the OMI group than in the non-OMI group (29.6% vs. 34.0%). Regarding mortality, in-hospital mortality, including hopeless discharges, was significantly lower in the OMI group than in the non-OMI group (1.8% vs. 3.1%).

An in-depth analysis of OMIs based on injury severity is presented in Table 2. Regarding age groups, riders aged 20–29 years had the highest proportion of non-severe injuries (25.5%), while elderly riders aged >65 years had the highest proportion of severe injuries (29.1%). Compared to the rate of non-severe injuries, the rate of severe injuries was higher during the day (55.7% vs. 50.8%) and was lower during the evening (31.0% vs. 38.2%). Motorcycle riders traveling on narrow alleys or farm roads were more likely to suffer severe injuries (14.2% vs. 9.1%), and head-on collisions were the most common collision pattern (9.2%).

**Table 2:** General characteristics, injury detail, and clinical results of occupational motorcycle injury cases in the study period, according to severity.

		Non-severe OMI (N = 7276)		Severe OMI (N = 1615)		Total OMI		
		N	%	N	%	N		p-value
Age	Median (Q1-Q3)	36 (24–52)		49 (29–68)		38 (24–55)		<0.001
Age group	<20	910	12.5	155	9.6	1065	12.0	<0.001
	20s	1858	25.5	253	15.7	2111	23.7	
	30s	1274	17.5	199	12.3	1473	16.6	
	40s	1128	15.5	209	12.9	1337	15.0	
	50s	949	13.0	233	14.4	1182	13.3	
	60–65	304	4.2	96	5.9	400	4.5	
	>65	853	11.7	470	29.1	1323	14.9	
Sex	Male	6732	92.5	1462	90.5	8194	92.2	0.007
	Female	544	7.5	153	9.5	697	7.8	
Injury time of the day	Day (07–18)	3696	50.8	900	55.7	4596	51.7	<0.001
	Evening (18–24)	2780	38.2	500	31.0	3280	36.9	
	Night (00–07)	800	11.0	215	13.3	1015	11.4	
ED arrival route	119 ambulance	4276	58.8	928	57.5	5204	58.5	<0.001
	Other ambulance	565	7.8	520	32.2	1085	12.2	
	Foot or own car	2398	33.0	154	9.5	2552	28.7	
	Other transport	37	0.5	13	0.8	50	0.6	
Place	Highway/motorway	359	4.9	83	5.1	442	5.0	<0.001
	Common road	6099	83.8	1271	78.7	7370	82.9	
	Alley/farm road	663	9.1	229	14.2	892	10.0	
	Other	155	2.1	32	2.0	187	2.1	
Collision pattern	Head on	582	8.0	148	9.2	730	8.2	<0.001
	Side-to-side	650	8.9	99	6.1	749	8.4	
	Rear-end	126	1.7	38	2.4	164	1.8	
	Capsized	482	6.6	84	5.2	566	6.4	
	Combination	312	4.3	39	2.4	351	3.9	
	Other	141	1.9	106	6.6	247	2.8	
Injury counterpart	Bicycle/motorcycle	269	3.7	46	2.8	315	3.5	<0.001
	Small vehicles, ≥ 4 wheels	3753	51.6	659	40.8	4412	49.6	
	Large vehicles	282	3.9	100	6.2	382	4.3	
	Fixed object	184	2.5	53	3.3	237	2.7	
	No counterpart	1424	19.6	299	18.5	1723	19.4	
	Human/animal	52	0.7	16	1.0	68	0.8	
	Other	28	0.4	10	0.6	38	0.4	
Alcohol	Yes	165	2.3	98	6.1	263	3.0	<0.001
Helmet	Yes	4890	67.2	765	47.4	5655	63.6	<0.001
TBI, TSI, or facial injury		897	12.3	473	29.3	1370	15.4	<0.001
TBI		321	4.4	376	23.3	697	7.8	<0.001
TSI		274	3.8	101	6.3	375	4.2	<0.001
ED LOS (hour), Median (Q1-Q3)		2.6 (1.4–4.8)		5.8 (3.3–12.1)		3.0 (1.6–5.7)		<0.001
Admission		1572	21.6	1056	65.4	2628	29.6	<0.001
Hospital LOS (day), Median (Q1-Q3)		12.0 (6.3–21.1)		15.4 (8.3–27.0)		13.4 (7.0–23.7)		<0.001
Overall mortality + hopeless discharge		55	0.8	101	6.3	156	1.8	<0.001

OMI; occupational motorcycle injury, ED; emergency department, TBI; traumatic brain injury, TSI; traumatic spine injury, ED; emergency department, LOS; length of stay. Note that severity is determined by excess mortality ratio-adjusted injury severity score over 24 points.

Factors associated with severe injuries among patients with OMIs are shown in Table 3. After adjustment, increasing age (AOR, 1.02; 95% CI, 1.01–1.02), driving under the influence (AOR, 1.90; 95% CI, 1.12–3.23), and not wearing a helmet (AOR, 1.57; 95% CI, 1.24–1.99) were significantly associated with severe injuries. Regarding collision counterparts, compared with small vehicle collisions, colliding with a human or an animal was associated with a significantly higher risk of injury severity (AOR, 3.33; 95% CI, 1.40–7.92).

**Table 3:** Adjusted odds ratios and confidence intervals of injury characteristics to severity among occupational motorcycle injury cases.

		OR	95% CI		AOR	95% CI	
Sex	Male vs. female	0.77	0.64	0.93	0.75	0.45	1.24
Age	by 1 year	1.03	1.02	1.03	1.02	1.01	1.02
Injury time	Night vs day	0.74	0.66	0.83	1.27	1.00	1.61
	Dawn vs day	1.10	0.93	1.31	1.61	1.12	2.31
ED arrival route, vs 119 ambulance	Other ambulance	4.24	3.69	4.87	3.81	2.86	5.06
	Foot or own car	0.30	0.25	0.35	0.30	0.21	0.44
	Other transport	1.62	0.86	3.06	1.36	0.43	4.33
Place, vs common road	Highway/motorway	1.11	0.87	1.42	1.15	0.81	1.63
	Alley/farm road	1.66	1.41	1.95	0.84	0.53	1.31
	Other	0.99	0.67	1.46	0.53	0.26	1.09
Collision pattern, vs head-on	Side-to-side	0.60	0.45	0.79	0.61	0.45	0.82
	Rear-end	1.19	0.79	1.78	0.96	0.62	1.51
	Capsized	0.69	0.51	0.92	0.97	0.56	1.70
	Combined collision	0.49	0.34	0.72	0.52	0.35	0.77
	Other	2.96	2.17	4.03	2.36	1.59	3.50
Counterpart, vs small vehicles, ≥ 4 wheels	Bicycle + motorcycle	0.97	0.71	1.35	1.43	0.86	2.39
	Large vehicles	2.02	1.58	2.57	1.47	0.98	2.20
	Fixed object	1.64	1.20	2.25	0.73	0.38	1.42
	No counterpart	1.20	1.03	1.39	0.83	0.50	1.39
	Human/animal	1.75	1.00	3.09	3.33	1.40	7.92
	Other	2.03	0.98	4.21	1.07	0.37	3.15
Alcohol	Yes vs no	3.11	2.37	4.09	1.90	1.12	3.23
Helmet	No vs yes	1.98	1.75	2.25	1.57	1.24	1.99

AOR; adjusted odds ratio, CI; confidence interval, ED; emergency department, amb; ambulance. Note that severity is determined by excess mortality ratio-adjusted injury severity score over 24 points.

## Discussion

We aimed to find out the impact of OMIs by analyzing their characteristics with non-OMIs and determining factors associated with severity, using a large scale nationwide database of a developed country. Our study revealed several findings about OMI patterns. Regarding the time of the day, OMIs most frequently occurred during the daytime. Regarding age, the victims were mostly aged 20–29 years, and severe injuries most frequently occurred among elderly riders. Patients with OMIs wore helmets more frequently than did patients with non-OMIs. The most common collision types were head-on, side-to-side, and collisions with small vehicles. The injury severity rate was lower among patients with OMIs than it was among patients with non-OMI. Traumatic brain and spinal injuries were more common among patients with OMIs than in those without; overall mortality was lower in this the OMI group than in the non-OMI group.

In contrast to most developed countries, where deliveries are mostly done using cars and trucks [23, 24], the delivery of couriers and merchandise using motorcycles is still very common and increasing due to the pandemic and advances in technology in Korea [25]. Motorcycle delivery has some advantages, such as being easier to navigate through traffic congestions, being cheaper to operate than cars or trucks, and requiring fewer skills and pre-requisite certifications [5, 26, 27]. This explains why many younger adults and even teenagers are found among motorcycle injury victims, as illustrated by the trend presented in Fig 3. Our findings are consistent with those of other studies performed in Brazil and Kenya [14, 28]. Although da Silva et al. [20] described young age as a factor for serious injuries, our study showed that injury severity and mortality increased with age. This discrepancy could be due to differences in the study methods; as mentioned in the Introduction, most studies on OMIs are based on surveys or questionnaires rather than medical records. Moreover, these studies were conducted in developing countries where motorcycles are a common mode of transport for people [11–14, 18, 28].

Most injuries occurred at night. Based on the in-depth analysis by the hour (Fig 4), the highest proportion of injuries occurred during the early evening, from 17:00 to 20:00, and the lunch hours, from 12:00 to 13:00. This finding could be explained by the increase in service demand at such times such as food delivery during lunch hours and package courier delivery during late afternoon hours. This explanation also aligns with the background of this study, as discussed in the Introduction section.

Based on pre-hospital data, car insurance was the most common type of medical insurance in both groups. Riders of online courier services are often freelancers instead of employees, leading to a rise in the number of freelancers [25, 29]. This may result in a lack of reporting of workplace injuries or filling out paperwork for occupational health insurance. This could potentially impact patient outcomes as they or their family members may opt for limited treatments. The exact impact of this is unclear, but the aforementioned is our hypothesis.

A major factor associated with injury severity is helmet usage. The importance of helmets has been previously reported in many studies; here, we cofirmed this finding [8, 10, 30]. Regarding the willingness and tendency to wear helmets, those involved in OMIs wore helmets more often. Traumatic brain injuries (TBIs) were significantly higher in the severe OMI group, suggesting a protective effect of a helmet while riding a motorcycle. In previous single-center studies on general motorcycle injuries in Korea, regardless of occupational injury status, the rate of helmet use varied between 40% and 80% [8–10, 26, 31]. This rate is higher than that in other developing countries where rates as low as 20%-30% have been reported [12, 28]. A study conducted in Brazil specified that the type of helmet is also important [14]. However, we were not able to distinguish the helmet types in our study.

Injury severity and mortality were lower among patients with OMIs than among those with non-OMIs. Compared to the findings of other motorcycle-relate studies, one of our unique findings is that there were higher TBI and TSI rates in patients with OMIs despite the lower severity and mortality compared to those in patients with non-OMIs. These results were observed with lower alcohol consumption and helmet usage rates, despite higher TBI or TSI rates in the OMI group. As we have stated in the Introduction, no previous studies directly compare OMIs to non-OMIs. Therefore, one of the possibilities we may consider is the ‘healthy worker effect’ or ‘healthy user bias’ [32, 33]. In fact, the working population tends to be ‘healthier,’ possibly resulting in lower mortality or morbidity than the general population when faced with a certain exposure. People who work tend to be more employed and use motorcycles (“healthy hire effect”), and people with health problems will be less likely to ride motorcycles (“healthy worker survivor effect”). Similarly, there were younger motorcycle drivers in the OMI group in our study. A worldwide report published by the World Health Organization describes elderly drivers are “more likely to be killed or seriously disabled than younger people” in a motor vehicle accident because of resilience [34]. We could not collect ‘traffic’ factors such as motorcycle speed or type of helmet used, which may influence TBI incidence. We found a higher rate of OMIs in highways, alleys, or collisions with large vehicles, which may contribute to the higher TBI or TSI. Furthermore, we hypothesize that many occupational motorcycle riders have more experience in riding and are, thus, more likely to suffer less severe injuries. Non-occupational motorcycle riding largely involves recreational driving. In addition, less riding under the influence (of alcohol) and the higher use of protective equipment may have contributed to lower injury severity and mortality among patients with OMIs. These results emphasize the importance of enforcing regulation about alcohol and protective equipment, in developing countries where motorcycle is still largely used for work or service purposes.

To reduce severe motorcycle injuries and mortality, besides using protective gear, the following measures are recommended: establishing a standard riding protocol, promoting safe driving, selecting appropriate equipment, offering proper compensation, and avoiding unreasonable delivery time goals [29]. Another recent growing trend is freelance car delivery of larger merchandise, similar to what occurs in the US and Europe [1, 25, 29, 35]. This trend may lead to less severe motorcycle injuries and reduce overall mortality.

This study has several limitations. First, although we were able to collect data on whether the driver was working, it was impossible to collect specific information on the occupation and tasks of the motorcycle rider. Therefore, we could not clarify whether the driver was delivering merchandise or riding a motorcycle for other occupational purposes. If such data points were available, we would have been able to distinguish both situations. Second, we could not determine whether the motorcycle injury occurred in an urban or rural area. This is because the occupational use of motorcycles differs in metropolitan, rural, and pastoral areas. Third, there were discrepancies in how the participating hospitals entered data into the database. Some variables would have been useful for additional analysis, such as disability or previous drug use; however, we had to exclude some variables with many “unknown” responses. Fourth, although the EDISS data are national, most hospitals participating in the EDISS are university institutions. Many trauma patients get transferred to smaller hospitals after initial surgery or critical care. Therefore, such patients’ outcomes were not considered since smaller long-term care facilities do not participate in the EDISS. If clinical outcomes after the interhospital transfer had been collected, our results would have more accurately reflected the actual mortality. Fifth, describing accident models is a important factor in analyzing traffic accidents. This includes using traffic flow model or Baysian analysis [36, 37]. Integration with traffic data may strengthen the value of nationwide clinical data in analyzing traffic accidents. However, EDISS data we obtained did not contain sufficient information to implement such modeling process.

In conclusion, patients with OMIs had higher helmet usage rates, lower injury severity, lower in-hospital mortality, and a lower admission rate than did patients with non-OMIs. The injury severity was associated with the time of the injury, colliding with other living objects, driving under the influence, and helmet usage. Our findings may help implementing proper regulation of motorcycle riding environment including protective equipment and promoting safe driving.
